# Gami–Chunggan Formula Prevents Motor Dysfunction in MPTP/p-Induced and A53T α-Synuclein Overexpressed Parkinson’s Disease Mouse Model Though DJ-1 and BDNF Expression

**DOI:** 10.3389/fnagi.2019.00230

**Published:** 2019-08-28

**Authors:** Sora Ahn, Quan Feng Liu, Jae-Hwan Jang, Jeonghun Park, Ha Jin Jeong, Youngman Kim, Dong-hee Kim, Guwon Jeong, Seung Tack Oh, Seong-Uk Park, Seung-Yeon Cho, Hi-Joon Park, Songhee Jeon

**Affiliations:** ^1^Department of Korean Medical Science, Graduate School of Korean Medicine, Kyung Hee University, Seoul, South Korea; ^2^Department of Neuropsychiatry, Graduate School of Oriental Medicine, Dongguk University, Gyeongju, South Korea; ^3^Department of Biomedical Sciences, Center for Creative Biomedical Scientists, Chonnam National University, Gwangju, South Korea; ^4^Research Institute, Dong Kwang Pharmaceutical Co., Ltd., Seoul, South Korea; ^5^Stroke and Neurological Disorders Center, Kyung Hee University Hospital at Gangdong, Kyung Hee University, Seoul, South Korea; ^6^Studies of Translational Acupuncture Research (STAR), Acupuncture and Meridian Science Research Center (AMSRC), Kyung Hee University, Seoul, South Korea

**Keywords:** Parkinson’s disease, traditional medicine, Chunggan extract, chronic disease model, mouse

## Abstract

The Gami–Chunggan formula (GCF) is a modification of the Chunggan (CG) decoction, which has been used to treat movement disorders such as Parkinson’s disease (PD) in Traditional East Asian Medicine. To evaluate the neuroprotective effects of GCF in chronic PD animal models, we used either a 5-week treatment of 1-methyl-4-phenyl-1,2,3,6-tetrahydropyridine with probenecid (MPTP/p) or the α-synuclein A53T overexpressed PD mouse model. C57BL/6 mice were treated with MPTP, in combination with probenecid, for 5 weeks. GCF was administered simultaneously with MPTP injection for 38 days. The A53T α-synuclein overexpressed mice were also fed with GCF for 60 days. Using behavioral readouts and western blot analyses, it was observed that GCF prevents motor dysfunction in the MPTP/p-induced and A53T α-synuclein overexpressed mice. Moreover, GCF inhibited the reduction of dopaminergic neurons in the substantia nigra (SN) and fibers in the striatum (ST) against MPTP/p challenge. The expression of DJ-1 was increased but that of α-synuclein was decreased in the SN of PD-like brains by GCF administration. *In vitro* experiments also showed that GCF inhibited 6-OHDA-induced neurotoxicity in SH-SY5Y neuroblastoma cell lines and that it did so to a greater degree than CG. Furthermore, GCF induced BDNF expression through phosphorylation of Akt, ERK, CREB, and AMPK in the SN of PD-like brains. Therefore, use of the herbal medicine GCF offers a potential remedy for neurodegenerative disorders, including Parkinson’s disease.

## Introduction

Parkinson’s disease (PD) is a chronic neurodegenerative disorder that is predominantly characterized by three representative motor features: akinesia, stiffness, and tremors (Conley and Kirchner, 1999; Kalia and Lang, 2015). Although the exact pathogenic mechanism of PD remains unknown, one study with PD patients suggested that oxidative stress and inflammatory pathways together induce apoptosis of dopaminergic neurons, eventually leading to manifestation of the disease (Hartmann, 2004). In addition, several genes have been correlated with the development of PD (de Silva et al., 2000). One among them is the presynaptic protein α-synuclein, a fibrillar factor of Lewy bodies that is linked to neuropathological features of PD; several different missense mutations of α-synuclein such as A53T, A30P, and E46K have been linked to early onset PD (Polymeropoulos et al., 1997; Krüger et al., 1998). Overexpression of wild-type or mutant A53T human α-synuclein in mice causes human PD-like symptoms such as neuronal degeneration and movement impairments (van der Putten et al., 2000).

Several toxins or reagents that mimic Parkinsonism both *in vitro* and *in vivo* have been reported in recent times, such as 1-methyl-4-phenyl-1,2,3,6-tetrahydropyridine (MPTP), 6-hydroxydopamine (6-OHDA), and rotenone (Beal, 2001). MPTP specifically targets dopaminergic neurons and causes severe and irreversible PD-like syndrome in non-human primates and humans. These subjects display biochemical and pathological hallmarks of PD (Przedborski et al., 2000) such as the obvious loss of dopaminergic neurons, astrogliosis, and activated microglia in the substantia nigra pars compacta (SNpc) (Beal, 2001). A second reagent, probenecid, can accelerate the mitochondrial toxicity of MPTP by interfering with ATP metabolism (Alvarez-Fischer et al., 2013). In the chronic MPTP/probenecid (MPTP/p) model, approximately 40–45% of dopaminergic neurons in the SNpc are lost within 3 weeks of treatment while 25% are lost in subchronic models without probenecid (Petroske et al., 2001; Meredith et al., 2008). In either case, death of dopaminergic neurons continues for at least 6 months, unlike in the subchronic and acute MPTP models (Petroske et al., 2001; Meredith et al., 2008). 6-OHDA, on the other hand, mimics symptoms of PD by generating free radicals after it is transported by the dopamine transporter, resulting in the cell death of dopaminergic neurons.

Treatment for PD typically comprises L-3,4-dihydroxyphenylalanine (L-dopa), a dopamine precursor and/or a dopamine agonist. Although this can reduce symptoms of PD, long-term use of the drug reduces its effectiveness and does not in fact stop disease progression (Kostic et al., 1991).

Chunggan (CG) extract has been used for the treatment of motor-related disorders, such as PD, in traditional oriental medicine. It includes six herbs: *Paeonia lactiflora* root, *Angelica gigas* root, *Bupleurum falcatum Linne* root, *Ligusticum chuanxiong* root, *Gardenia jasminoides Ellis* fruit, and *Paeonia suffruticosa Andrews* root bark. We have previously presented evidence for the pharmaceutical effects and mechanism of action of modified CG extract and its combination with L-dopa in the MPTP-induced PD model (Ahn et al., 2017; Chang et al., 2018). Although we already demonstrated the pharmacological properties of CG on acute PD symptoms, we did not examine its effects in a chronic disease model. Therefore, in this study, MPTP/p or α-synuclein A53T overexpression was used to establish a chronic PD mouse model. To improve the treatment efficacy of CG, a modified formula named Gami–Chunggan formula (GCF) was prepared, consisting of CG plus the *Syzygium aromaticum* bud and the *Agastache rugosa O. Kuntze* herb that has strong radical scavenging activities (data not shown). We aim to demonstrate the effect and mechanisms of action of GCF on PD-like phenotypes such as motor symptoms and neuroprotection in the chronic MPTP/p-induced or α-synuclein A53T overexpression induced PD mice models.

## Materials and Methods

### Apparatus, Chemicals, and Reference Compounds

All analytical experiments were conducted with the Shimadzu LC-20AD XR High Performance Liquid Chromatography (HPLC) system and an SPD-M20A Photo Diode Array (PDA) detector (Kyoto, Japan). Acetonitrile and ethanol were obtained from J.T. Baker (PA, United States), and 18.2 MΩ distilled water was purified using Younglin’s Aqua Max Ultra 370 (Anyang, South Korea) series. Geniposide, paeoniflorin, tilianin, paeonol, eugenol, saikosaponin A, ligustilide and decursin reference compounds were used for HPLC analysis and all were purchased from ChemFaces (Hubei, China).

### Preparation of GCF and CG Extract

All packages of *P. lactiflora* root, *L. chuanxiong* root, *A. gigas* root, *B. falcatum Linne* root, *G. jasminoides Ellis* fruit, *S. aromaticum* bud, *P. suffruticosa Andrews* root bark, and *A. rugosa O. Kuntze* herb were purchased from the Tae-won-dang herb supplier (Daegu, South Korea). The origins of all plant batches were confirmed and deposited at Dongkwang Pharmaceutical Research and Development Center for extraction and HPLC analysis. For extraction of GCF, air-dried *P. lactiflora* root (60 g), *L. chuanxiong* root (40 g), *A. gigas* root (40 g), *B. falcatum Linne* root (32 g), *G. jasminoides Ellis* fruit (16 g), *S. aromaticum* bud (60 g), *P. suffruticosa Andrews* root bark (16 g), and *A. rugosa O. Kuntze* herb (40 g) were uniformly mixed and 30% ethanol (3.24 L) added to make a 30% ethanol mixture (10% w/v). For preparation of CG extract, *S. aromaticum* bud and *A. rugosa O. Kuntze* herb were excluded from GCF. After heating was initialized under reflux and the temperature reached 95 °C, the 30% ethanol mixture was extracted for a further 4 h. The 30% ethanol extract was cooled down for 30 min and filtered with Whatman #2 filter paper. The filtered extract was freeze dried to obtain GCF dried extract powder. The extraction was repeated 10 times.

### Preparation of Standards and Samples

For standardization of GCF extract, The Korean Pharmacopoeia and scientific papers were reviewed and its major components identified (Yun et al., 2008; Tuan et al., 2012; Korean Food and Drug Administration [KFDA], 2015; Baek et al., 2016). One reference compound from each plant component of GCF was selected as a standard; these were geniposide, paeoniflorin, paeonol, eugenol, saikosaponin A, and decursin. For HPLC analysis, each reference compound was dissolved and mixed thoroughly to make stock solution. Individual stock solutions were added in uniform amounts to make working standard mixtures, which were used for the simultaneous separation and determination of compounds. For HPLC analysis, 1 g of freeze-dried GCF extract powder was weighed and added into a 10 mL volumetric flask with HPLC-grade 70% ethanol as solvent. The GCF extract powder was further extracted using an ultra-sonicator for 1 h. After sonication, the extract was filtered and used as GCF extract sample for HPLC analysis.

### HPLC Analysis of GCF

All experiments were conducted with the Shimadzu LC-20AD XR HPLC system. GCF extract samples were analyzed under the developed HPLC method and the reference compounds in GCF extract samples were quantified using Shimadzu’s Lab Solutions software. Chromatographic separation was accomplished by using a YMC Pack Pro C18 (250 × 4.6 mm, 5 μm) column (YMC Company, Japan) with a flow rate of 1.0 mL/min at 30°C. To optimize detection, the entire UV spectrum of each reference compound was reviewed at different wavelengths. For optimum analysis, we selected 210 nm for saikosaponin A and ligustilide, and 230 nm for geniposide, paeoniflorin, tilianin, paeonol, eugenol and decursin.

### MPTP Mouse Model

Male C57BL/6 mice (Central Laboratories Animal Inc., South Korea) with a mean weight of 29.5 g were reared under standard conditions. The experimental processes were approved by the Institutional Animal Treatment Ethical Committee at the Dongguk University Campus (No. 2017-0992) and followed NIH guidelines. Mice were administered with intraperitoneal (i.p.) injections of saline, MPTP (30 mg/kg, dissolved in saline; Sigma-Aldrich, MO, United States), or L-3,4-dihydroxyphenylalanine (L-dopa, Sigma-Aldrich, MO, United States) for 6 days. Mice were allocated to six groups:

(1) Control (saline-injected group, *n* = 5)
(2) MPTP (MPTP + intraorally saline-treated group, *n* = 5)
(3) MPTP + GCF 100 (MPTP + intraorally 100 mg/kg of GCF-treated group, *n* = 5)
(4) MPTP + GCF 200 (MPTP + intraorally 200 mg/kg of GCF-treated group, *n* = 5)
(5) MPTP + GCF 300 (MPTP + intraorally 300 mg/kg of GCF-treated group, *n* = 5)
(6) MPTP + L-dopa (MPTP + intraperitoneally 10 mg/kg of L-dopa-treated group, *n* = 5)

Gami–Chunggan formula or L-dopa treatment was given simultaneously with MPTP injection for 14 days. Behavior tests were done on day 15.

### MPTP/p Mouse Model

Protocols for mouse experiments were revised and permitted by the Institutional Animal Care and Use Committee at the Dongguk University Campus (No. 2017-025) and followed NIH guidelines. Mice, excluding the control group, were administered MPTP (25 mg/kg in saline, i.p.) along with probenecid (100 mg/kg in 5% NaHCO_3_, i.p.) (MPTP/p). These mice were treated with 10 injections of MPTP with probenecid, every 3.5 days for 5 weeks. During the schedule, two of them died after the ninth and tenth injection in the MPTP/p and MPTP/p + GCF (100 mg/kg) groups, respectively. Mice were allocated to five groups:

(1) Control (saline-injected group, *n* = 8)
(2) MPTP/p (MPTP/p + intraorally saline-treated group, *n* = 9)
(3) MPTP/p + GCF 100 (MPTP/p + intraorally 100 mg/kg of GCF -treated group, *n* = 9)
(4) MPTP/p + GCF 200 (MPTP/p + intraorally 200 mg/kg of GCF treated group, *n* = 9)
(5) MPTP/p + GCF 300 (MPTP/p + intraorally 300 mg/kg of GCF-treated group, *n* = 9)

Gami–Chunggan formula was administered intraorally along with MPTP/p injections. Each dose of GCF was dissolved in 0.9% saline and administered once a day for 38 days. The drugs were administered each day at 2:30 p.m.

### Transgenic Mice

83Vle mice (Prnp-SNCA^∗^A53T) with a B6C3H background (Jackson Laboratory, Bar Harbor, Maine, United States) were bred at the Dongguk University and animal protocols followed previously described methods (Lee et al., 2017) (No. 2017-0992). A53T hemizygous mice (*n* = 22) at 13–14 months of age were divided into three groups:

(1) Control (intraorally saline-treated group, *n* = 7)
(2) GCF 100 (intraorally 100 mg/kg of GCF -treated group, *n* = 7)
(3) GCF 300 (intraorally 300 mg/kg of GCF-treated group, *n* = 8)

Saline or GCF was orally administered every day for 60 days. Behavior experiments were done on day 60 and the mice were then sacrificed. The feed efficiency ratio (FER) was calculated as total increased weight divived by the total amount of food consumption.

### Biochemical Analysis of Blood

For biochemichal blood analysis, total blood was collected with heparin-syringe tubes and centrifuged at 3000 rpm for 15 min at 4°C. Plasma was collected and kept at −70°C. Glucose, total cholesterol, high-density lipoprotein (HDL) cholesterol, GOT (Glutamate Oxaloacetate Transaminase)/GPT (Glutamate Pyruvate Transaminase), and triglyceride (TG) were examined with analysis kits (Asan Pharmaceutical, Seoul, South Korea).

### Akinesia

Akinesia was measured as the latency in time taken to move four limbs. The test was administered as previously described (Ahn et al., 2017). The test was repeated four times for each animal.

### Catalepsy

Catalepsy was recorded as the time period for which animals retained their front legs, once placed, on a bar suspended above the floor of the test apparatus (Ahn et al., 2017). The time point at which the mice lifted their front paws from the bar marked the end of the time period. This experiment was repeated four times for each animal and mean value was calculated.

### Rotarod Test

The rotarod test was used to assess neurological impairment such as motor coordination and balance. The experimental procedure followed was as previously reported (Ahn et al., 2017).

### Pole Test

We performed a pole test 60 days after GCF administration, using an instrument 55 cm in height and 1.3 cm in diameter. The mice were held by their tails, with their heads positioned upward near the top of the pole and their forepaw on top of the pole. The time taken by the mouse to fall fully head down (orient down time) and the time taken to reach the bottom (transverse down time) were recorded. Mice were adjusted to the task by performing five trials per day for 3 days before the behavior test.

### Brain Immunohistochemistry

Brain tissue preparation and immunohistochemistry methods were performed as per a previous report (Ahn et al., 2017). Briefly, sectioned slices were incubated with rabbit anti-tyrosine hydroxylase (1:1000; Santa-Cruz Biotechnology, TX, United States) overnight at room temperature. They were then stained using ABC methods (Vectastain Elite ABC kit; Vector Laboratories, Inc., CA, United States) and developed with diaminobenzidine (Sigma, MO, United States). The sections were mounted, coverslipped, and imaged using a light microscope (BX51; Olympus Japan Co., Tokyo, Japan).

### Western Blot Analysis

The substantia nigra (SN) and striatum (ST) from mice brains were isolated and homogenized with RIPA buffer (Thermo Fisher Scientific, MA, United States). Supernatant from these lysates were mixed with 4X Laemmli’s sample buffer and boiled at 99°C for 5 min. The samples were electrophoresed through 10% or 15% Tris-SDS-PAGE and then transferred to an Immobilon-P membrane (Millipore, MA, United States). The blotted membrane was blocked with 5% skim milk in Tris-Buffered Saline containing 0.05% Tween 20 (TBS-T buffer) for 1 h. After washing the membrane with TBS-T, each primary antibody was added and incubated overnight at 4°C. The primary antibodies used were α-synuclein (1:500; Cell Signaling, MA, United States), p-Akt (1:1000; Cell Signaling), Akt (1:1000; Cell Signaling), p-ERK (1:1000; Cell Signaling), ERK (1:1000; Cell Signaling), p-CREB (1:1000; Cell Signaling), CREB (1:1000; Cell Signaling), BDNF (1:500; Santa-Cruz Biotechnology, TX, United States), p-AMPK (1:1000; Cell Signaling), AMPK (1:500; Cell Signaling), DJ-1 (1:1000; Cell Signaling), p-synapsin-1 (1:1000; Cell Signaling), Synapsin-1 (1:1000; Cell Signaling), β-actin (1:4000; Sigma, MO, United States), TNF-α (1:500; Cell Signaling), BCL-2 (1:1000; Cell Signaling) and BAX (1:500; Abcam, Cambridge, MA). The blots were mixed with HRP-conjugated secondary rabbit (Thermo Fisher Scientific, MA, United States) or mouse (Thermo Fisher Scientific) antibodies. Bands were detected using the ChemiDoc XRS + (Bio-Rad, CA, United States) and analyzed using Image Lab software (ver. 2.0.1; Bio-Rad).

### Cell Culture

The human neuroblastoma SH-SY5Y cell line was purchased from the American Type Culture Collection (Rockville, MD, United States) and maintained in DMEM containing 10% FBS and 1% antibiotics (Hyclone Laboratories Inc., UT, United States). Serum-deprived cells were treated with GCF for 30 min and then stressed with 200 μM of 6-OHDA (Biosource International, CA, United States) for 24 h. To examine cell viability, 100X of 3-(4,5-dimethylthiazol-2-yl)-2,5-diphenyltetrazolium bromide (MTT) (Sigma) was added to the media and the cells were incubated for 3 h at 37°C in a CO_2_ incubator. The media was then removed and remaining dye in the cells solubilized with DMSO. The optical density of each well was calculated using a spectrophotometer (Versamax microplate reader, Molecular Device, CA, United States) at a wavelength of 570 nm.

### Statistical Analyses

GraphPad Prism (ver. 5; GraphPad Software, Inc., CA, United States) was used to perform all statistical analyses. One-way ANOVA was used to analyse the data excluding AIMs. Two-way ANOVA was used in AIMs data considering time and group as factors. All data are expressed as means ± SEM. *p* < 0.05 were considered as statistically significant.

## Results

### Development of the Chemical Profile of GCF and Identification of Major Components

**FIGURE 1:**
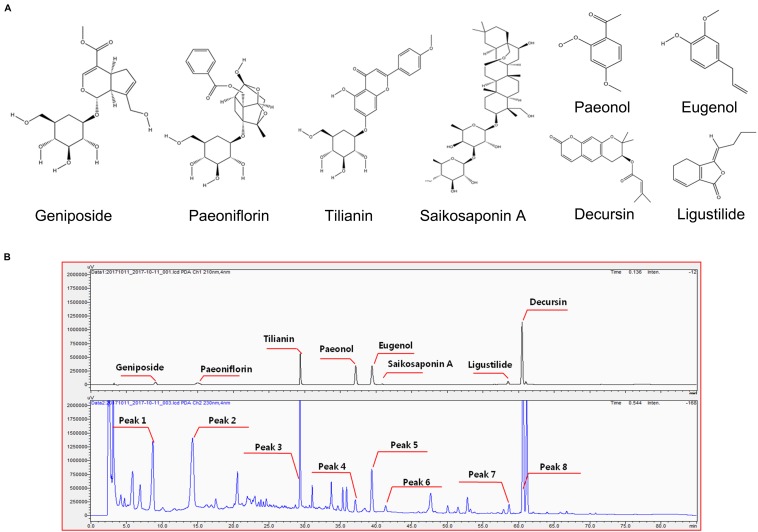
Chemical structure of reference compounds and HPLC chromatograms of GCF samples. **(A)** Chemical structures of the reference compounds were drawn using ChemDraw software. **(B)** Eight reference compounds were selected, and their retention times observed using the Shimadzu PDA system (upper panel). Eight peaks with same retention time were identified from GCF extracts (lower panel).

Plants contain hundreds of constituents, some of which are present at very low concentrations. Compositions may vary within different batches of plant material due to factors like freshness, temperature, light, water, time of collection, drying, and storage methods. To minimize this variation, the origins and suppliers of plant materials and the chemical compositions of GCF extract powder were controlled and standardized. One reference compound from each plant source used in GCF was selected for standardization based a thorough literature scan (Figure 1A; Yun et al., 2008; Tuan et al., 2012; Korean Food and Drug Administration [KFDA], 2015; Baek et al., 2016). Eight reference compounds were analyzed using the developed HPLC method; all of them were well-resolved and detected in the chromatograms (Figure 1B). The retention times of these compounds were used for identification of those from the GCF extract samples. When the GCF extract samples were analyzed, their chromatograms showed eight peaks matching those of the eight reference compounds (Figure 1B). The composition of the eight compounds in GCF extract powder are geniposide (13.97 ± 0.03 mg/g), ligustilide (0.8 ± 0.12 mg/g), tilianin (6.23 ± 0.03 mg/g), eugenol (12.13 ± 0.03 mg/g), saikosaponin A (1.1 ± 0.04 mg/g), paeoniflorin (19.26 ± 0.27 mg/g), paeonol (0.97 ± 0.08 mg/g), and decursin (11.20 ± 0.14 mg/g). No significant differences were found between batches.

### GCF Has Neuroprotective Effects on 6-OHDA-Induced Apoptosis in SH-SY5Y Cells

**FIGURE 2:**
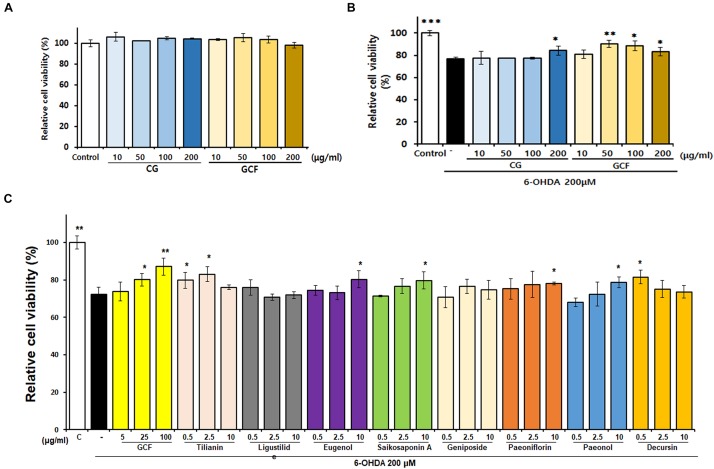
Neuroprotective effects of GCF and its active compounds on SH-SY5Y cells. **(A)** Effect of CG and GCF extracts on viability of SH-SY5Y cells. SH-SY5Y cells were treated with CG and GCF of different concentrations and relative cell viability examined using the MTT assay. **(B)** Neuroprotective effect of CG and GCF extract on 6-OHDA-treated SH-SY5Y cells. **(C)** Neuroprotective effect of GCF on 6-OHDA-treated SH-SY5Y cells. Cell viability was determined using the MTT assay. Data are presented as mean ± SEM (^∗∗∗^*p* < 0.001, ^∗∗^*p* < 0.01, ^∗^*p* < 0.05 compared with the MPTP group; *n* = 3).

The cytotoxic effect of GCF was tested using the MTT assay (Figure 2A) (*F*_(__8__,__22__)_ = 3.15, *p* < 0.0205). When administered at a concentration of 200 μg/ml, neither CG nor GCF showed any cytotoxicity in SH-SY5Y cells. Treatment with 6-OHDA at a concentration of 200 μM for 24 h reduced cell viability to 76.81 ± 1.53% (*n* = 3) of the control value (100 ± 2.26%, *n* = 3) (*F*_(__9__,__25__)_ = 14.82, *p* < 0.0001) (Figure 2B). However, SH-SY5Y cell viability of groups treated with 200 μg/ml CG recovered to 83 ± 2.55% (*n* = 3) and those of groups treated with 50 μg/ml GCF increased to 95.70 ± 1.98% (*n* = 3) (Figure 2B). These observations indicated that GCF has a stronger protective effect than CG against 6-OHDA toxicity in SH-SY5Y cells. To compare the neuroprotective effects of each component GCF, SH-SY5Y cells were pre-treated with different concentrations of the components or of GCF itself, followed by treatment with vehicle or 200 μM 6-OHDA for 24 h (*n* = 3). Among the components, tilianin (2.5 μg/ml, 83.00 ± 4.15%), eugenol (10 μg/ml, 86.21 ± 3.99%), saikosaponin A (10 μg/ml, 79.71 ± 4.55%), paeoniflorin (10 μg/ml, 77.92 ± 0.76%), paeonol (10 μg/ml, 78.62 ± 2.97%), and decursin (0.5 μg/ml, 81.52 ± 3.65%) facilitated significant recovery of the 6-OHDA-treated SH-SY5Y cells. (*F*_(__28__,__85__)_ = 7.36, *p* < 0.0010) (Figure 2C).

### GCF Inhibits MPTP-Induced Movement Impairments

**FIGURE 3:**
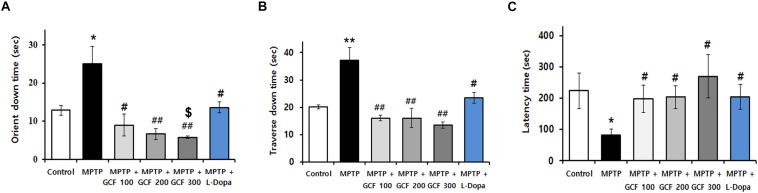
Effects of GCF on MPTP-induced motor behavior dysfunctions and dopamine levels in the ST. Pole test of MPTP-injected PD model **(A)** orient down time and **(B)** transverse down time. **(C)** Latency time in overall rod performance test of MPTP-injected PD model. MPTP: MPTP only; GCF 100: GCF 100 mg/kg; GCF 200: GCF 200 mg/kg; GCF 300: GCF 300 mg/kg. Data are expressed as mean ± SEM (^∗^*p* < 0.05, ^∗∗^*p* < 0.01 compared to control group; ^#^*p* < 0.05, ^##^*p* < 0.01 compared to MPTP group; ^$^*p* < 0.05 compared to L-dopa group; *n* = 5).

To verify the protective effect of GCF in a PD animal model, GCF or L-dopa was administered to MPTP-treated mice for 14 days. MPTP induced severe motor impairments in the pole and rotarod tests when compared to the control group. Administration of GCF, however, significantly reduced the orient down time (*F*_(__5__,__35__)_ = 8.88, *p* < 0.0001), traverse down time (*F*_(__5__,__34__)_ = 6.81, *p* < 0.0002) and increased the time taken to fall from the rod (*F*_(__5__,__35__)_ = 3.04, *p* < 0.224). L-dopa-treated mice were used as positive controls and they also recovered movement impairments, but not to the extent of the GCF-treated group (Figures 3A–C).

### Preventive Effect of GCF on MPTP/p-Induced Movement Impairments and Dopaminergic Neuron Loss

**FIGURE 4:**
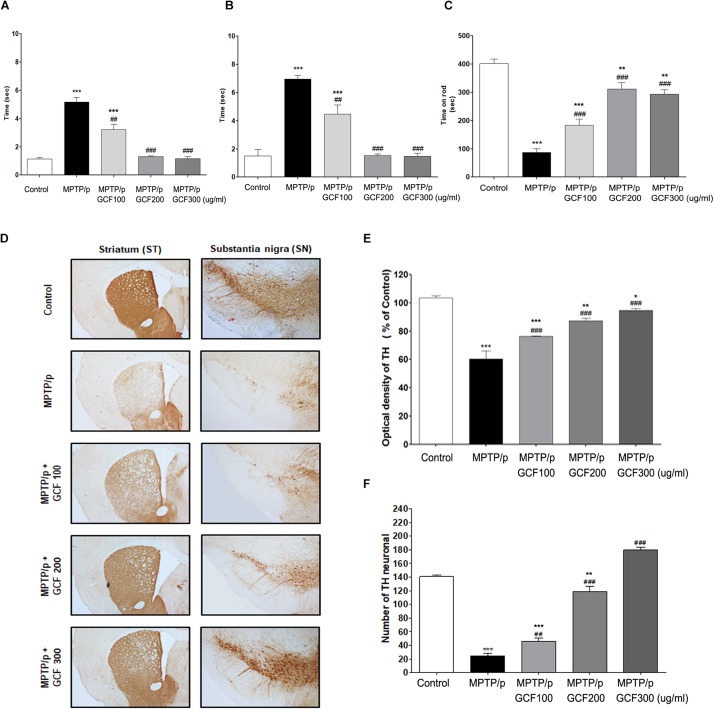
Effects of GCF on MPTP/p-induced motor behavior dysfunctions and other neuroprotective effects. **(A)** Akinesia, **(B)** catalepsy, and **(C)** rotarod test were measured at day 36 post MPTP/p administration in control (*n* = 8), MPTP/p (*n* = 9), MPTP/p + GCF 100 (*n* = 9), MPTP/p + GCF 200 (*n* = 9), and MPTP/p + GCF 300 (*n* = 9) mice. **(D)** Brain sections were stained immunohistochemically for TH-positive dopaminergic fibers in the ST and neurons in the SN (scale bar, ST – 200 μm; SN – 100 μm). **(E)** The optical density of TH-positive dopaminergic fibers in the ST is shown in bar graphs. **(F)** Total TH-positive cell numbers in the SN. MPTP/p: MPTP/probenecid; GCF 100: GCF 100 mg/kg; GCF 200: GCF 200 mg/kg; GCF 300: GCF 300 mg/kg. Data are expressed as mean ± SEM (^∗^*p* < 0.05, ^∗∗^*p* < 0.01, ^∗∗∗^*p* < 0.001 compared to control group; ^##^*p* < 0.01, ^###^*p* < 0.001 compared to MPTP/p group).

To verify the neuroprotective effects of GCF against motor symptoms, behavioral experiments were conducted on the chronic MPTP/p mouse model. Chronic administration of MPTP/p failed to initiate movement (akinesia, *F*_(__4__,__38__)_ = 159.30, p < 0.0001) or correct an externally enforced posture (catalepsy, *F*_(__4__,__38__)_ = 248.00, *p* < 0.0001) when compared to the control group (*p* < 0.0001 and *p* > 0.0001, respectively). Administration of GCF significantly diminished MPTP/p-induced akinesia and catalepsy (MPTP vs. GCF 100, *p* < 0.01; GCF 200 and 300, *p* < 0.001 each) (Figures 4A,B). In the rotarod test (*F*_(__4__,__38__)_ = 43.60, *p* < 0.0001), the MPTP/p group dropped from the rod at a significantly higher rate than the control group (*p* < 0.001) (Figure 4C), but the GCF-treated group remained on the rod longer than the MPTP/p group (MPTP/p vs. GCF 100, 200, and 300, *p* < 0.001 in all cases), indicating that GCF can reduce MPTP/p-induced hypokinesia.

To examine the effect of GCF on MPTP/p-induced dopaminergic neuronal loss, tyrosin hydroxylase (TH) was stained in the ST and SNpc of mice brains (SN: *F*_(__4__,__15__)_ = 189.80, *p* < 0.0001; ST: *F*_(__4__,__15__)_ = 36.86, *p* < 0.0001). In the MPTP/p group, the number of TH-positive cells in both the ST and SNpc was decreased and fiber density was lower than that of the control group (*p* < 0.001 and *p* < 0.001, respectively) (Figures 4D–F). However, GCF-treated mice (all doses) showed increased numbers of TH-immunopositive cells in the SNpc (MPTP vs. GCF 100, *p* < 0.01; GCF 200 and 300, *p* < 0.001 each) and denser fibers in the ST than those of the MPTP/p group (MPTP/p vs. GCF 100, 200 and 300, each *p* < 0.001) (Figures 4D–F). These data are indicative of the protective effect GCF on MPTP/p-induced dopaminergic neuron loss.

### Suppressive Effect of GCF on the Level of α-Synuclein and TNF-α in the MPTP/p PD Model

**FIGURE 5:**
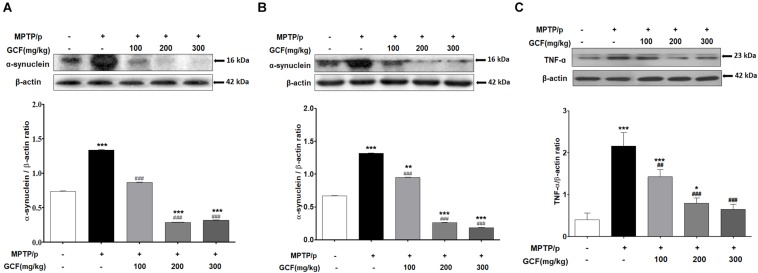
Expression of α-synuclein and TNF-α in MPTP/p-induced mouse brains. Immunoblots assessing the effect of GCF treatment on levels of α-synuclein in **(A)** ST and **(B)** SN and **(C)** the levels of TNF-α in SN of MPTP/p induced mice. Data are expressed as mean ± SEM, (^∗^*p* < 0.05, ^∗∗^*p* < 0.01, ^∗∗∗^*p* < 0.001 compared to control group; ^##^*p* < 0.01, ^###^*p* < 0.001 compared to MPTP/p group; *n* = 4).

The levels of α-synuclein, a Lewy Body marker, were assessed by immunoblotting. Chronic MPTP/p treat m ent significantly enhanced α-synuclein expression in the SN and ST (SN: *F*_(__4__,__15__)_ = 8666.00, *p* < 0.0001; ST: *F*_(__4__,__15__)_ = 3672.00, *p* < 0.0001) when compared to vehicle treatment (*p* < 0.001, respectively) (Figures 5A,B). GCF treatment, on the other hand, significantly and dose-dependently suppressed the MPTP/p-induced α-synuclein expression in both the SN and ST (MPTP/p vs. GCF 100, 200 and 300, *p* < 0.001 in all cases) (Figures 5A,B). We also examined the levels of tumor necrosis factor α (TNF-α), a cytokine involved in the inflammatory response and regulation of immune cells, in the SN of chronic MTPT/p mice. The expression level of TNF-α (*F*_(__4__,__15__)_ = 19.80, *p* < 0.0001) was significantly increased in the chronic MPTP/p group when compared to the control group (*p* < 0.001). However, treatment with GCF significantly reduced the levels of TNF-α (MPTP vs. GCF 100, *p* < 0.01; GCF 200 and 300, *p* < 0.001 each) (Figure 5C).

### Changes in Protein Expression Upon GCF Administration in the MPTP/p PD Model

**FIGURE 6:**
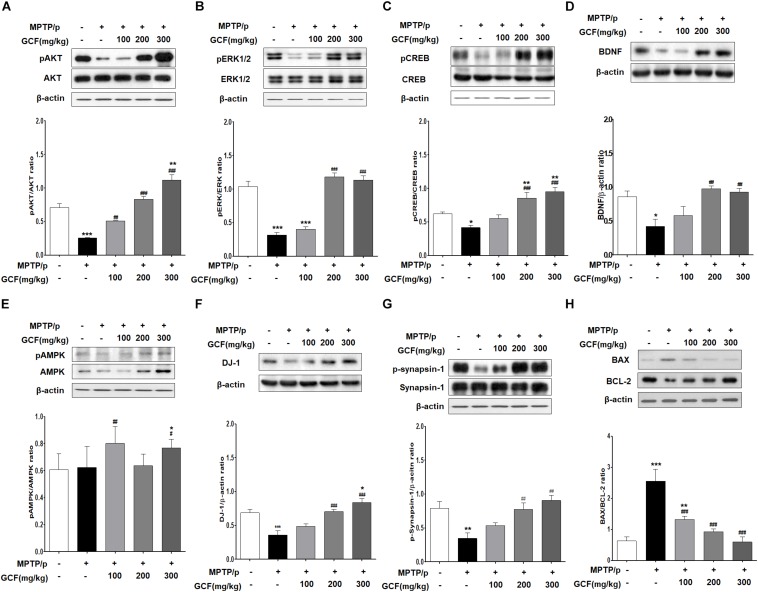
Mechanisms of protective effects of GCF in MPTP/p-induced PD mouse model. Immunoblots of SN extracts probed using the following antibodies: **(A)** pAkt/Akt/β-actin, **(B)** pERK/ERK/β-actin, **(C)** pCREB/CREB/β-actin, **(D)** BDNF/β-actin, **(E)** pAMPK/AMPK/β-actin, **(F)** DJ-1/β-actin, **(G)** pSynapsin-1/Synapsin-1/β-actin, and **(H)** BAX/BCL-2/β-actin. The intensities of protein bands were quantitated by densitometry and the bands indicating phosphorylated protein were normalized against either the total protein or β-actin. Data are expressed as mean ± SEM (^∗^*p* < 0.05, ^∗∗^*p* < 0.01, ^∗∗∗^*p* < 0.001 compared to control group; ^##^*p* < 0.01, ^###^*p* < 0.001 compared to MPTP/p group; *n* = 4).

Chronic MPTP/p treatment induced a marked reduction in Akt activation (*F*_(__4__,__15__)_ = 46.65, *p* < 0.0001) and extracellular signal-regulated kinase (ERK) activity (*F*_(__4__,__15__)_ = 48.68, *p* < 0.0001) in comparison to the control group (*p* < 0.001) (Figures 6A,B). Conversely, GCF (200 and 300 mg/kg) significantly blocked the Akt and ERK dephosphorylation seen in the chronic MPTP/p-treated animals (*p* < 0.001 each) (Figures 6A,B). In addition, chronic MPTP/p treatment reduced the activation of cAMP response element binding (CREB) (*F*_(__4__,__15__)_ = 14.93, *p* < 0.0001) and decreased the expression of brain-derived neuroprotective factor (BDNF) (*F*_(__4__,__15__)_ = 6.76, *p* < 0.0064) when compared to controls (*p* < 0.001 each) (Figures 6C,D). GCF (200 and 300 mg/kg) also significantly inhibited CREB dephosphorylation and BDNF down-regulation (CREB: *p* < 0.001 each; BDNF: *p* < 0.01 each) (Figures 6C,D). Interestingly, the phosphorylation of ERK and expression of BDNF were more dramatic at 200 mg/kg than at 300 mg/kg.

Next, we examined the phosphorylation level of adenosine monophosphate (AMP)-activated protein kinase (AMPK) as a negative regulator of autophagy. Chronic MPTP/p treatment inhibited phosphorylation of AMPK (*F*_(__4__,__15__)_ = 17.38, *p* < 0.0001) (Figure 6E), but treatment with GCF increased AMPK phosphorylation (Figure 6E). Notably, total AMPK levels (*F*_(__4__,__15__)_ = 19.80, *p* < 0.0001) were also increased by GCF treatment (MPTP vs. GCF 100, *p* < 0.01; GCF 300, *p* < 0.05) (Figure 6E). Furthermore, when DJ-1 expression was investigated, we found that it was significantly reduced in the chronic MPTP/p group (*F*_(__4__,__15__)_ = 14.67, *p* < 0.0001) when compared to the control group (*p* < 0.001) or GCF-treated group (200 and 300 mg/kg, *p* < 0.001) (Figure 6F). The phosphorylation level of synapsin-1 (*F*_(__4__,__15__)_ = 7.35, *p* < 0.0017) was also lower in the chronic MPTP/p group than in the control group (*p* < 0.01), whereas GCF treatment significantly inhibited synapsin-1 dephosphorylation in a dose-dependent manner (*p* < 0.01) (Figure 6G).

We then investigated whether the expression of BAX, a critical downstream mediator of apoptosis belonging to the BCL-2 family, was affected by GCF treatment. To check this, we determined the expression levels of the anti-apoptotic protein BCL-2 and apoptotic protein BAX. In the chronic MPTP/p group, the level of BCL-2 (*F*_(__4__,__15__)_ = 19.80, *p* < 0.0001) was significantly lower than that of the control group (*p* < 0.001), but GCF treatment inhibited BCL-2 downregulation (MPTP vs. GCF 100, *p* < 0.01; GCF 200, 300, *p* < 0.001) (Figure 6H). BAX levels were increased by chronic MPTP/p treatment (*F*_(__4__,__15__)_ = 19.80, *p* < 0.0001), but GCF administration blocked this increase (MPTP vs. GCF 100, *p* < 0.01; GCF 200, 300, *p* < 0.001) (Figure 6H). When we compared the ratio of BAX to BCL-2 (*F*_(__4__,__15__)_ = 19.80, *p* < 0.0001), we found that it was higher in chronic MPTP/p treated mice by 2.5 fold, but reduced in the SN of GCF-treated mice in a dose-dependent manner (*p* < 0.001) (Figure 6H).

### Neuroprotective Effect of GCF on A53T α-Synuclein Tg Mice

**FIGURE 7:**
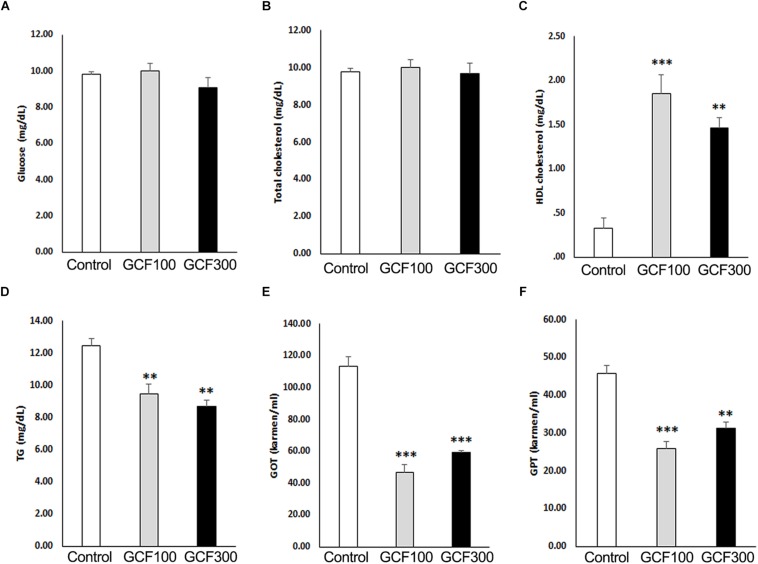
Effect of GCF on plasma biochemistry of A53T α-synuclein Tg mice. **(A)** Glucose, **(B)** total cholesterol, **(C)** HDL cholesterol, **(D)** triglyceride (TG), **(E)** GOT, and **(F)** GPT levels were analyzed from plasma. The data are expressed as mean ± SEM (^∗∗^*p* < 0.01, ^∗∗∗^*p* < 0.001 compared to control group; *n* = 6).

Recent studies have shown that high blood levels of triglyceride (TG) and LDL-cholesterol have been associated with rapid cognitive decline (Ma et al., 2017). Therefore, we examined the biochemical parameters of plasma obtained from GCF-treated A53T α-synuclein Tg mice. As shown in Figure 7, GCF-treated Tg mice showed significantly higher levels of plasma HDL-cholesterol (*F*_(__2__,__13__)_ = 5.70, *p* < 0.0001; 100 mg/kg, *p* < 0.001; 300 mg/kg, *p* < 0.01) and significantly lower levels of plasma TG (*F*_(__2__,__13__)_ = 1.50, *p* < 0.0087), GOT (*F*_(__2__,__13__)_ = 12.56, *p* < 0.0009) and GPT (*F*_(__2__,__13__)_ = 0.75, *p* < 0.0001) when compared to control Tg mice (*p* < 0.01). However, the levels of glucose (*F*_(__2__,__13__)_ = 2.07, *p* < 0.1606) and total cholesterol (*F*_(__2__,__13__)_ = 2.52, *p* < 0.1140) were similar in both groups.

**FIGURE 8:**
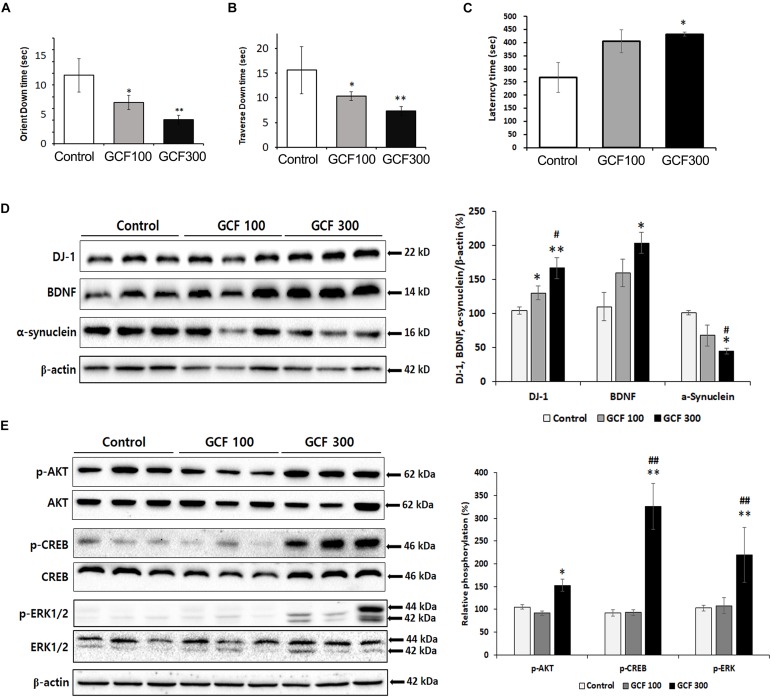
Neuroprotective effect of GCF on A53T α-synuclein Tg mice. **(A)** Orient down time and **(B)** transverse down time of pole test, and **(C)** latency time of overall rotarod performance were measured in each group. The lysates of SN were electrophoresed and immunoblotted with each antibodies against α-synuclein, DJ-1, BDNF, and the phosphorylated forms of Akt, CREB, and ERK. The intensity of each band was normalized to that of β-actin **(D)** or total form and β-actin **(E)** and presented in bar graphs. The data are expressed as mean ± SEM (^∗^*p* < 0.05, ^∗∗^*p* < 0.01 compared to control group; ^#^*p* < 0.05 compared to GCF 100 mg/kg treated group; *n* = 7).

To assess the efficacy of GCF on motor symptoms, we conducted behavior tests on A53T α-synuclein Tg mice. In the pole test, GCF showed a dose-dependent reduction of the orient down time (*F*_(__2__,__7__)_ = 3.27, *p* < 0.0857) and traverse down time (*F*_(__2__,__7__)_ = 3.00, *p* < 0.1043) when compared to control Tg mice (each *p* < 0.001) (Figures 8A,B). Additionally, in the rotarod test we observed significant improvement of the latency time (*F*_(__2__,__7__)_ = 4.54, *p* < 0.0431) in GCF 300 treated mice in comparison to the control mice (*p* < 0.01) (Figure 8C).

A53T α-synuclein Tg mice showed no differences in TH expression in the SN as compared to control mice (Supplementary Figure S1). This is in agreement with previous reports showing that, in transgenic mice expressing A53T α-synuclein under the tyrosine hydroxylase promoter, the number of nigral neurons and levels of striatal dopamine are unchanged relative to wild-type mice for up to 1 year (Matsuoka et al., 2001). It has also been shown that the total number of SN neurons is not changed between the Tg and wild-type mice (Jia et al., 2018), indicating that dopaminergic cell loss has not yet occurred in the Tg mice. Despite lacking these markers of PD, the A53T mutation was identified in familial cases leading to early onset of parkinsonian symptoms (Athanassiadou et al., 1999), as well as in cases of motor impairment in mice (Uchihara and Giasson, 2016).

Expression of DJ-1 is known to clear the accumulation and toxicity of α-synuclein (Zhou et al., 2011; Zondler et al., 2014). GCF treatment induced DJ-1 (*F*_(__2__,__4__)_ = 24.69, *p* < 0.0013; 100 mg/kg, *p* < 0.05; 300 mg/kg, *p* < 0.01) and BDNF (*F*_(__2__,__4__)_ = 13.53, *p* < 0.0260; 300 mg/kg, *p* < 0.05) expression in a dose-dependent fashion when compared to control. It also decreased α-synuclein expression (*F*_(__2__,__4__)_ = 22.04, *p* < 0.0160; 300 mg/kg, *p* < 0.05) in the SN of A53T α-synuclein Tg mice (Figure 8D). Moreover, the GCF 300 group showed significant phosphorylation of Akt (*F*_(__2__,__4__)_ = 39.24, *p* < 0.0400), CREB (*F*_(__2__,__4__)_ = 62.68, *p* < 0.00129), and ERK (*F*_(__2__,__4__)_ = 9.81, *p* < 0.0010) in the SN of A53T α-synuclein Tg mice (*F*_(__2__,__4__)_ = 13.53, *p* < 0.0260; 300 mg/kg, *p* < 0.05) (Figure 8E). These data suggest that GCF treatment may decrease α-synuclein expression through DJ-1 expression and exercise a neuroprotective effect through BDNF expression.

## Discussion

In this study, we demonstrated that administration of GCF significantly improves behavioral impairments in A53T α-synuclein overexpressed mice and blocks the loss of doparminergic neurons in MPTP/p-induced mice, both of which are models of chronic Parkinson’s disease. In addition, GCF induces BDNF and DJ-1 expression in the SN and downregulates α-synuclein in PD models.

The CG decoction has been used to improve motor function of PD patients in traditional oriental medicine. GCF is a modification of the CG decoction, consisting of some herbal components in addition to the standard CG mixture. Among the GCF components, paeoniflorin shows the most prominent anti-PD activities (Li et al., 2013). Paeoniflorin comes from the *P. lactiflora* root, which has been used to treat neurodegenerative disorders like PD in traditional medicine clinics. Paeoniflorin has been known to rescue MPP^+^ and acidic damage-induced PC12 cell apoptosis through the autophagic pathway (Cao et al., 2010). This treatment also alleviates neurological deficits associated with unilateral striatal 6-OHDA lesion PD models (Liu et al., 2007). It attenuates neuroinflammation and dopaminergic neurodegeneration in PD mouse models by activation of the adenosine A1 receptor (Liu et al., 2006). Geniposide, an active element of *G. jasminoides Ellis*, has tranquilizing effects and is an important herb used in Traditional Chinese Medicine for dementia (Su et al., 2016). It shows neuroprotective effects by suppressing α-synuclein expression (Su et al., 2016), induces growth factors and reduces apoptosis in PD models (Chen et al., 2015). Eugenol, a phenol extracted from cloves, is an antioxidant (Ito et al., 2005), monoamine oxidase (MAO) inhibitor (Tao et al., 2005), and displays other neuroprotective properties in PD models (Kabuto and Yamanushi, 2011). *A. rugosa*, a medicinal herb of the family *Lamiaceae* native to China, Korea, and Japan, has been used to decrease nausea and vomiting and treat fungal infections. Pharmacological studies have found that *A. rugosa* and one of its components, tilianin, have antiviral (Wang et al., 2009), antimicrobial (Shin, 2004), antioxidant (Guo et al., 2011), cardiovascular (Yuan et al., 2017), anti-inflammatory, anti-diabetic, and anti-hyperlipidemic properties (García-Díaz et al., 2016).

In the present study, we examined six active components (geniposide, paeoniflorin, tilianin, eugenol, saikosaponin A, and decursin) from the GCF mixture using *in vitro* assays, and for the first time demonstrated that tilianin has anti-PD activity in SH-SY5Y neuroblastoma cells. In addition, we showed that GCF has a stronger neuroprotective effect on the neuroblastoma cells than CG or its individual components (Figure 2C), suggesting that GCF might be a good formulation to treat PD patients. One possible caveat of this study is that we treated the cells first with GCF and then with 6-OHDA. The antioxidant properties of GCF may have inhibited the oxidation of 6-OHDA, thus weakening its toxicity toward the cells. However, GCF administered at a high concentration of 200 ug/ml did not show cytotoxicity and inhibited the cell death induced by 6-OHDA. This indicated that it most likely did not suppress the oxidation of 6-OHDA, but had a genuine protective effect on the cells.

cAMP response element binding is a transcription factor stimulated by Ser-133 phosphorylation, and it has numerous downstream effecters: protein kinase C (PKC), protein kinase A (PKA), ERK1/2, and several PI3K/Akt/GSK-3β pathways (Adams and Sweatt, 2002; Carlezon et al., 2005). In this study, we found that GCF treatment increased the levels of CREB phosphorylation in the SN of MPTP/p-treated and A53T a-synuclein Tg mice. An upstream activator of CREB, Akt, was phosphorylated by GCF treatment as well. The observed ERK phosphorylation, however, may be attributed not just to GCF treatment, but to increased proinflammatory cytokine release, which could be neurotoxic in itself (Hua et al., 2002). In addition to these elements, administration of GCF was shown to suppress TNF-α expression (Figure 5C). These alterations in protein expression indicate that GCF activates Akt, which in turn enhances CREB activation and exerts neuroprotective effects in the PD model mice.

DJ-1, a sensor of oxidative stress (Canet-Avilés et al., 2004), can decrease the accumulation and toxicity of α-synuclein in PD models (Shendelman et al., 2004; Zhou et al., 2011; Sun et al., 2012; Zondler et al., 2014; Lee et al., 2017). It is also known to activate ERK and Akt pathways to induce cell proliferation and survival (Oh and Mouradian, 2017). In this study, we demonstrated that GCF activates Akt, which might in turn be due to the induction of DJ-1 expression. The final cellular output of these signaling pathways is the reduction of α-synuclein accumulation, relieving the symptoms of PD in animal models.

MPTP-induced oxidative stress induces apoptosis through the activation of BCL-2 family proteins, including anti-apoptotic BCL-2 and pro-apoptotic BAX (Yang et al., 1997; Crompton, 2000). It has been reported that DJ-1 translocates to the mitochondria and binds to BCL-X_L_ in response to UV-B irradiation, inhibiting both rapid degradation of BCL-X_L_ and mitochondrial apoptosis (Ren et al., 2011). Specifically, BCL-X_L_ interacts with BAX to block its oligomerization in the mitochondrial membrane, thereby protecting cells from BAX-induced mitochondrial membrane permeabilization (Yin et al., 1994). In the present study, it was shown that GCF induces DJ-1, which may suppress BCL-2 degradation and BAX expression, resulting in the inhibition of MPTP-induced apoptosis in PD models.

Autophagy is the process of degradation and elimination of aggregated proteins. Inhibition of this process induces neuronal degeneration in the central nervous system (Hara et al., 2006; Komatsu et al., 2006). AMPK activation is linked to the maintenance of autophagy (Mihaylova and Shaw, 2011) and neurogenesis (Dagon et al., 2005). AMPK activity in dopaminergic neurons has also been shown to be necessary for neuroprotection in a mouse model of PD (Bayliss et al., 2016). In addition, neuronal AMPK is part of an important signaling pathway that regulates BDNF, an essential mediator of neurogenesis (Kim and Leem, 2016; Liu et al., 2014). In this context, we demonstrated that treatment with GCF activates AMPK and induces BDNF expression in MPTP/p-induced PD mice models. Thus, this study provides evidence for the neuroprotective effects of GCF through AMPK activation in the SN.

We conducted both *in vitro* and *in vivo* efficacy tests of GCF in SH-SY5Y cells and mouse models. However, we primarily focused on the *in vivo* test to determine the effective doses of GCF in humans. We found that the effective doses in mice were 200 mg/kg and 300 mg/kg and the Human Equivalent Doses (HED) (Reagan-Shaw et al., 2008) were the following: 972.9730 mg/day (200 mg/kg × (3/37) × 60 kg) and 1459.4595 mg/day (300 mg/kg × (3/37) × 60 kg], respectively. In line with this observation, we conducted a 13-week oral toxicity study in Sprague–Dawley (SD) rats to determine the No Observed Adverse Effect Level (NOAEL). Based on this oral toxicity study, the NOAEL was assessed to be 2000 mg/kg/day by the KFDA-certified Good Laboratory Practice (GLP) Contract Research Organization (CRO), ChemOn (Yongin-Si, Gyeonggi, South Korea). The concentrations of GCF that were used in mice are therefore safe for therapeutic usage (data not shown).

Generally, when animals are tested for therapeutic effects of new compounds, the drug is administered simultaneously with disease induction. Therefore in this experiment, medication in the form of GCF was administered at the same time as MPTP, implying that the protective effects of GCF in the MPTP/p study are due to blockage of MPTP metabolism to MPP^+^. However, we also examined the neuroprotective effects of GCF after PD induction in the MPTP PD models (data not shown) and in the A53T α-synuclein Tg animal model, and found similar effects to those seen with simultaneous GCF administration. Hence, it can be inferred that GCF is an effective reagent for neuroprotection and regeneration when administered to PD patients.

In this study, we compared the anti-PD effects of GCF with L-dopa in the MPTP PD model (Figure 3). Although L-dopa did not recover the loss of dopaminergic cells, it did improve behavioral impairment, which is in concert with previous reports that L-dopa improves abnormal behavior in Parkinson’s patients by supplementing deficient dopamine. However, longterm use of L-dopa may induce certain complications such as eventual loss of symptom control, leading to dyskinesia (Hauser, 2009). We found that the effect of GCF was more significant than that of L-dopa in terms of motor control (Figure 3), suggesting that GCF may normalize the function of dopaminergic cells in MPTP-treated mice. However, further studies are essential to determine the levels of dopamine and its metabolites in order to fully illustrate the protective effects of GCF.

In summary, the present findings demonstrate the neuroprotective effect of GCF against MPTP- or MPTP/p-induced motor deficits and dopaminergic cell death. In addition, GCF administration diminishes behavioral impairments in α-synuclein A53T overexpressed mice. GCF activates elements of cell survival pathways such as Akt, ERK, and CREB in PD models and induced DJ-1 and BDNF expression. Moreover, GCF decreases α-synuclein expression and pro-apoptotic BAX expression through DJ-1 induction in chronic PD models. Therefore, the use of GCF, a herbal medicine, could be a potential remedy for neurodegenerative disorders such as Parkinson’s disease.

## Ethics Statement

The experimental processes were approved from the Institutional Animal Treatment Ethical Committee, Dongguk University Campus (Nos. 2017-0992, 2017-025, and 2017-0992) and followed the NIH guidelines.

## Author Contributions

All authors were responsible for the study concept and design. SA, J-HJ, and JP carried out the immunoblotting of animal experiments in MPTP/p PD model. QL and HJ carried out the cell study, MPTP PD model, and α-synuclein PD animal experiments. YK, DK, GJ, and SO participated in the extraction of herbal medicine and analytical experiments. S-UP and S-YC organized the oriental medicine prescription. H-JP and SJ conceived the study and wrote the draft manuscript.

## Conflict of Interest Statement

The authors declare that the research was conducted in the absence of any commercial or financial relationships that could be construed as a potential conflict of interest.

## Supplementary Material

FIGURE S1 Tyrosin hydroxylase expression in the SN of WT and A53T α-synuclein Tg mice. **(A)** The lysates of SN were electrophoresed and immunoblotted with TH or β-actin antibody. The intensity of each band was normalized to that of β-actin and presented in bar graphs **(B)**. The data are expressed as mean ± SEM.
