# Supplementary figures and images for: Gami–Chunggan Formula Prevents Motor Dysfunction in MPTP/p-Induced and A53T α-Synuclein Overexpressed Parkinson’s Disease Mouse Model Though DJ-1 and BDNF Expression
Source: Front Aging Neurosci. 2019 Aug 28;11:230. doi: 10.3389/fnagi.2019.00230 (PMC6724569; doi:10.3389/fnagi.2019.00230)

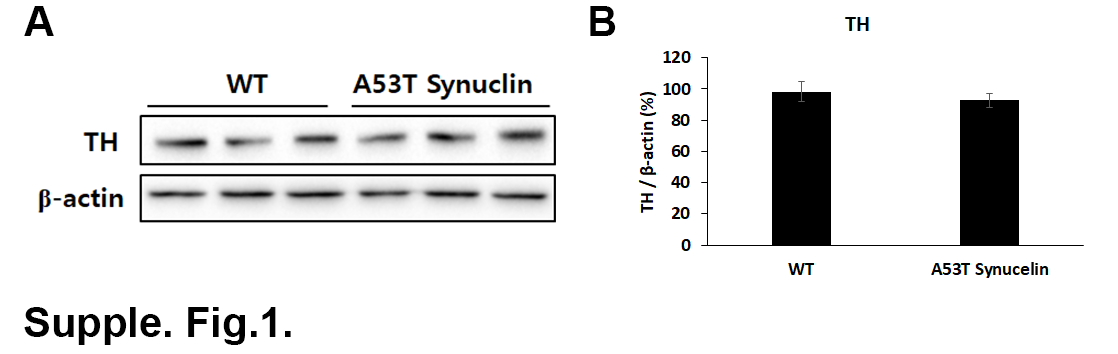

Supplement: FIGURE S1 — Tyrosin hydroxylase expression in the SN of WT and A53T α-synuclein Tg mice. (A) The lysates of SN were electrophoresed and immunoblotted with TH or β-actin antibody. The intensity of each band was normalized to that of β-actin and presented in bar graphs (B). The data are expressed as mean ± SEM. [file Image_1.TIF]
